# Association of Smoking Cessation and Survival Among Young Adults With Myocardial Infarction in the Partners YOUNG-MI Registry

**DOI:** 10.1001/jamanetworkopen.2020.9649

**Published:** 2020-07-08

**Authors:** David W. Biery, Adam N. Berman, Avinainder Singh, Sanjay Divakaran, Ersilia M. DeFilippis, Bradley L. Collins, Ankur Gupta, Amber Fatima, Arman Qamar, Josh Klein, Jon Hainer, Michael J. Blaha, Marcelo F. Di Carli, Khurram Nasir, Deepak L. Bhatt, Ron Blankstein

**Affiliations:** 1 Division of Cardiovascular Medicine, Department of Medicine, Brigham and Women’s Hospital, Harvard Medical School, Boston, Massachusetts; 2 Division of Cardiology, Department of Medicine, Yale University School of Medicine, New Haven, Connecticut; 3 New York Presbyterian/Columbia University Irving Medical Center, New York, New York; 4 Department of Radiology, Brigham and Women’s Hospital, Harvard Medical School, Boston, Massachusetts; 5 Department of Medicine, Tufts Medical Center, Boston, Massachusetts; 6 The Johns Hopkins Ciccarone Center for the Prevention of Cardiovascular Disease, Baltimore, Maryland; 7 Center for Outcomes Research, Houston Methodist, Houston, Texas

## Abstract

**Importance:**

Despite significant progress in primary prevention, the rate of myocardial infarction (MI) continues to increase in young adults.

**Objectives:**

To identify the prevalence of tobacco use and to examine the association of both smoking and smoking cessation with survival in a cohort of adults who experienced an initial MI at a young age.

**Design, Setting, and Participants:**

The Partners YOUNG-MI registry is a retrospective cohort study from 2 large academic centers in Boston, Massachusetts, that includes patients who experienced an initial MI at 50 years or younger. Smoking status at the time of presentation and at 1 year after MI was determined from electronic medical records. Participants were 2072 individuals who experienced an MI at 50 years or younger between January 2000 and April 2016. The dates of analysis were October to December 2019.

**Main Outcomes and Measures:**

Deaths were ascertained from the Social Security Administration Death Master File, the Massachusetts Department of Vital Statistics, and the National Death Index. Cause of death was adjudicated independently by 2 cardiologists. Propensity score–adjusted Cox proportional hazards modeling was used to evaluate the association between smoking cessation and both all-cause and cardiovascular mortality.

**Results:**

Among the 2072 individuals (median age, 45 years [interquartile range, 42-48 years]; 1669 [80.6%] men), 1088 (52.5%) were smokers at the time of their index hospitalization. Of these, 910 patients were further classified into either the cessation group (343 [37.7%]) or the persistent smoking group (567 [62.3%]) at 1 year after MI. Over a median follow-up of 11.2 years (interquartile range, 7.3-14.2 years), individuals who quit smoking had a statistically significantly lower rate of all-cause mortality (hazard ratio [HR], 0.35; 95% CI, 0.19-0.63; *P* < .001) and cardiovascular mortality (HR, 0.29; 95% CI, 0.11-0.79; *P* = .02). These values remained statistically significant after propensity score adjustment (HR, 0.30 [95% CI, 0.16-0.56; *P* < .001] for all-cause mortality and 0.19 [95% CI, 0.06-0.56; *P* = .003] for cardiovascular mortality).

**Conclusions and Relevance:**

In this cohort study, approximately half of individuals who experienced an MI at 50 years or younger were active smokers. Among them, smoking cessation within 1 year after MI was associated with more than 50% lower all-cause and cardiovascular mortality.

## Key Points

**Question:**

Is smoking cessation associated with lower mortality among young adults after an initial myocardial infarction (MI)?

**Findings:**

In this cohort study of 2072 individuals who experienced an initial MI at 50 years or younger, approximately half were smokers at the time of their MI, and 62% continued to smoke at 1 year after MI. Smoking cessation within 1 year after MI was associated with a statistically significant reduction in long-term all-cause and cardiovascular mortality.

**Meaning:**

Smoking cessation after MI was associated with a clinically significant reduction in all-cause and cardiovascular mortality in a cohort of patients who experienced an MI at a young age.

## Teaser

This cohort study identifies the prevalence of tobacco use and examines the association of both smoking and smoking cessation with survival in a cohort of adults who experienced a myocardial infarction at a young age.

## Introduction

The use of inhaled tobacco products is a strong risk factor for coronary artery disease (CAD), myocardial infarction (MI), and mortality.^1,2^ A combination of endothelial dysfunction, increased myocardial oxygen demand, and heightened risk of thrombosis are all believed to contribute to this pathophysiology.^3,4,5,6,7,8^ Fortunately, this elevated risk can be attenuated—if not largely reversed—by smoking cessation.^9,10,11,12,13,14,15,16,17,18,19,20,21,22,23^

Although prior research^13,17,18,20,21,23,24,25,26,27^ has consistently illustrated the benefits of smoking cessation on mortality in both the general population and in patients after MI, investigations have been limited to older patient populations. Accordingly, the benefits of smoking cessation after MI have not been well studied among younger patients with established CAD. In addition, past studies involving smokers have often yielded a phenomenon known as “the smoker’s paradox,” whereby those who smoked after MI were observed to have a lower average mortality risk than nonsmokers.^10,17,20,23,24,25,26,27^ Although this observation may be associated with a difference in age between smokers and nonsmokers—with nonsmokers being approximately 10 years older in many cohorts^17,20,21,24,25,26,27,28^—it is unknown if such a phenomenon arises when comparing younger populations of smokers and nonsmokers.

Considering that the rate of MI is increasing among younger individuals^29^ and that smoking in the United States is prevalent among these age groups,^30,31^ further research is needed to understand the potential benefits of smoking cessation in younger patient populations with known CAD. Therefore, the objectives of this study were to identify the prevalence of tobacco use and to examine the association of smoking and smoking cessation with survival in a cohort of adults who experienced an initial MI at a young age.

## Methods

### Study Population

The design of the Partners YOUNG-MI registry has been previously described.^32^ In brief, it is a retrospective cohort study from 2 large academic medical centers in Boston, Massachusetts (Brigham and Women’s Hospital and Massachusetts General Hospital), that included all consecutive patients who experienced an initial MI at 50 years or younger between January 2000 and April 2016. The dates of analysis were October to December 2019. All electronic medical records were adjudicated by a team of study physicians, as previously described.^32^ For the present analysis, only patients adjudicated to have had an MI caused by atherothrombotic CAD (type 1 MI) were included, as defined by the third universal definition of MI.^33^ Individuals with known CAD (defined as a prior MI or revascularization) were excluded from this analysis.

Study approval for the Partners YOUNG-MI registry was granted by the Institutional Review Board at Partners HealthCare along with a waiver of informed consent because of the retrospective study design. This study followed the Strengthening the Reporting of Observational Studies in Epidemiology (STROBE) reporting guideline.

### Risk Factors

For each individual, the presence of cardiovascular (CV) risk factors was ascertained through a detailed review of electronic medical records corresponding to the period up to and including the index admission. The following risk factors were evaluated: history of premature CAD in a first-degree relative, depression, anxiety, psychotic disorder, diabetes, hypertension, hyperlipidemia, obesity, alcohol use, illicit substance use, 10-year atherosclerotic CV disease (ASCVD) risk score,^34^ and Charlson Comorbidity Index (CCI).

The socioeconomic status of each patient was estimated by calculating a neighborhood stress score (NSS7), a composite measure of economic stress generated from census variables (with a higher score indicating increased financial hardship).^35^ In addition, invasive angiographic findings were ascertained through a review of all available cardiac catheterization images by a single interventional cardiologist blinded to all patient outcomes and risk factors. Detailed definitions of all risk factors, including how the CCI was calculated,^36^ as well as how the extent and severity of disease were quantified, are provided in eAppendix 1 in the Supplement and have been previously published.^32,37^

### Smoking Status

Smoking status of individuals at the time of the index hospitalization and at 1 year after MI was determined through a detailed review of electronic medical records by study investigators. At their index hospitalization, individuals were classified as never smokers, former smokers, or current smokers. Current smoking was defined as a documented history of smoking any type or amount of tobacco product within 1 month before admission. The duration, frequency, and the number of pack-years were captured whenever possible.

Those individuals classified as current smokers at their index hospitalization and who had available tobacco use data at 1 year after MI were further categorized as belonging to either the cessation group or the persistent smoking group. Patients within the cessation group were those who had documentation of sustained abstinence, defined as abstinence from inhaled tobacco for at least 3 months immediately before their 1-year follow-up period and without any documented relapse. Patients within the persistent smoking group were those who had reported continued tobacco use within the 1-year follow-up period without any indication of abstinence. Although our hospital system offers smoking cessation counseling to all individuals who are known tobacco users, these data were not adequately captured in the electronic medical records. Accordingly, data regarding smoking cessation counseling were not included in the data set.

### Outcomes

The outcomes of the study were all-cause mortality and CV mortality. Deaths were determined from the Social Security Administration Death Master File, the Massachusetts Department of Vital Statistics, and the National Death Index. Cause of death was adjudicated independently by 2 physicians (A.N.B., A.S., A.G., and A.Q.), as previously described.^32^ Death was classified as CV mortality^38^ or non-CV mortality. Causes of CV deaths included acute MI, heart failure, sudden cardiac death, ischemic stroke, nontraumatic hemorrhagic stroke, immediate complications of a CV procedure, CV hemorrhage, pulmonary embolism, and peripheral artery disease. Both death and cause of death were assessed with blinding to both smoking and cessation status.

### Propensity Score and Case Matching

To investigate the association of smoking cessation with long-term mortality, a propensity score was developed to measure the likelihood that a patient would quit tobacco use within 1 year of their index MI. We reviewed all baseline characteristics available at the time of the index admission for inclusion in a multivariable logistic regression analysis using smoking cessation within 1 year after MI as the dependent variable. Variables were selected for inclusion in the model if they were found to be potential confounders based on prior research^39,40,41^ or if they were statistically significantly predictive (*P* ≤ .05) of cessation within our cohort on a univariate basis. The association between each continuous variable and the log odds of smoking cessation were modeled using restricted cubic splines with 5 knots.^42^ eAppendix 2 in the Supplement describes the independent variables included in the model as well as the performance characteristics of the propensity score. After calculation of the propensity score, patients in the persistent smoking group were matched to patients in the cessation group on the basis of the propensity score using one-to-one matching with a caliper width equal to 0.2 of the SD of the logit of the propensity score (Table 1).^43^

**Table 1.** Comparison of Differences in Baseline Characteristics Between Full Propensity Score Sample and Propensity Score–Matched Sample

Baseline variable	Original sample			Matched sample		
	No./total No. (%)		Standardized difference	No./total No. (%)		Standardized difference
	Continued smoking (n = 540)	Quit smoking (n = 336)		Continued smoking (n = 309)	Quit smoking (n = 309)	
**Demographic characteristics**						
Age at the time of MI, mean (SD), y	44.0 (4.8)	43.9 (5.0)	0.014	43.9 (5.0)	44.0 (5.0)	0.009
Male sex	416 (77.0)	277 (82.4)	0.135	251 (81.2)	252 (81.6)	0.008
White race	413 (76.5)	248 (73.8)	0.062	241 (78.0)	228 (73.8)	0.098
Neighborhood stress score, mean (SD)	−0.30 (0.78)	−0.43 (0.75)	0.180	−0.45 (0.77)	−0.41 (0.74)	0.062
Insurance category^a^						
None	51/513 (9.9)	32/320 (10.0)	0.002	33/296 (11.1)	31/294 (10.5)	0.019
Public	180/513 (35.1)	84/320 (26.3)	0.192	89/296 (30.1)	83/294 (28.2)	0.040
Private	282/513 (55.0)	204/320 (63.8)	0.179	174/296 (58.8)	180/294 (61.2)	0.050
History of premature CAD in a first-degree relative	168 (31.1)	97 (28.9)	0.049	80 (25.9)	88 (28.5)	0.058
ST-elevation MI	340 (63.0)	190 (56.5)	0.131	181 (58.6)	182 (58.9)	<0.001
Segment Involvement Score, mean (SD)	2.75 (1.77)	3.05 (1.90)	0.166	3.00 (1.84)	2.97 (1.89)	0.017
Index LVEF, mean (SD), %	53 (11)	54 (12)	0.074	54 (11)	54 (12)	0.023
Depression	96 (17.8)	38 (11.3)	0.184	32 (10.4)	38 (12.3)	0.061
Anxiety	88 (16.3)	45 (13.4)	0.082	35 (11.3)	42 (13.6)	0.069
Psychotic disorder	27 (5.0)	6 (1.8)	0.178	5 (1.6)	6 (1.9)	0.024
Length of index hospital stay, mean (SD), d	4.6 (5.2)	4.6 (4.3)	0.006	4.7 (5.2)	4.6 (4.3)	0.010
**Risk factors**						
Diabetes	102 (18.9)	63 (18.8)	0.004	62 (20.1)	57 (18.4)	0.041
Hypertension	244 (45.2)	148 (44.0)	0.023	143 (46.3)	131 (42.4)	0.078
Hyperlipidemia	495 (91.7)	314 (93.5)	0.068	289 (93.5)	288 (93.2)	0.013
Obesity	170 (31.5)	149 (44.3)	0.267	127 (41.1)	124 (40.1)	0.020
Alcohol use	111 (20.6)	56 (16.7)	0.099	55 (17.8)	54 (17.5)	0.009
Illicit substance use	0.2 (0.4)	0.1 (0.3)	0.160	0.1 (0.3)	0.1 (0.3)	0.072
Angina	498 (92.2)	298 (88.7)	0.134	283 (91.6)	272 (88.0)	0.124
Peripheral vascular disease	8 (1.5)	11 (3.3)	0.117	6 (1.9)	11 (3.6)	0.100
ASCVD score, mean (SD)	9.2 (7.0)	9.1 (6.9)	0.021	9.6 (6.9)	9.0 (7.0)	0.087
Charlson Comorbidity Index score, mean (SD)	1.6 (1.1)	1.4 (0.7)	0.145	1.5 (0.9)	1.4 (0.7)	0.079
**Laboratory values, mean (SD)**						
Total cholesterol, mg/dL	193 (66)	192 (45)	0.010	197 (75)	192 (44)	0.086
HDL cholesterol, mg/dL	36 (9)	36 (9)	0.032	35 (9)	36 (9)	0.125
LDL cholesterol, mg/dL	121 (58)	119 (38)	0.051	121 (58)	119 (38)	0.051
Triglycerides, mg/dL	195 (152)	205 (168)	0.060	195 (152)	205 (168)	0.031
Creatinine, mg/dL	1.0 (0.3)	1.0 (0.3)	0.023	1.0 (0.3)	1.0 (0.3)	0.036
eGFR, mL/min/1.73 m^2^	89.8 (19.1)	89.5 (17.7)	0.014	91.1 (18.1)	89.9 (17.6)	0.070
**Patient management**						
Cardiac catheterization	530 (98.1)	333 (99.1)	0.082	307 (99.4)	306 (99.0)	0.036
Coronary revascularization	485 (89.8)	314 (93.5)	0.132	290 (93.9)	287 (92.9)	0.039
**Medical therapy at discharge**						
Statin	500 (92.6)	318 (94.6)	0.084	294 (95.1)	292 (94.5)	0.029
Aspirin	525 (97.2)	330 (98.2)	0.066	302 (97.7)	304 (98.4)	0.047
P2Y12 inhibitor	477 (88.3)	298 (88.7)	0.011	274 (88.7)	273 (88.3)	0.010
β-Blocker	497 (92.0)	322 (95.8)	0.159	296 (95.8)	295 (95.5)	0.016
ACE inhibitor/ARB	333 (61.7)	219 (65.2)	0.073	199 (64.4)	200 (64.7)	0.007
Diuretic	48 (8.9)	32 (9.5)	0.022	27 (8.7)	28 (9.1)	0.011

Abbreviations: ACE, angiotensin-converting enzyme; ARB, angiotensin II receptor blocker; ASCVD, atherosclerotic cardiovascular disease; CAD, coronary artery disease; eGFR, estimated glomerular filtration rate; HDL, high-density lipoprotein; LDL, low-density lipoprotein; LVEF, left ventricular ejection fraction; MI, myocardial infarction.

^a^ For the 833 patients (95.1%) and 590 patients (95.5%) with available insurance data, respectively.

### Statistical Analysis

All analyses were performed using Stata, version 15.1 (StataCorp). Categorical variables are reported as frequencies and proportions and were compared with χ^2^ test or Fisher exact test, as appropriate. Continuous variables are reported as means or medians and were compared with *t* test or Mann-Whitney test, as appropriate. The proportional hazards assumption was assessed by analyzing the Schoenfeld residuals. Survival curves were compared using the log-rank test.

Cox proportional hazards modeling was used to assess the association of baseline smoking and smoking cessation and to obtain corresponding hazard ratios (HRs) and 95% CIs for both all-cause and CV mortality. Patients were censored on the date of querying their source of vital statistics. In the analyses of CV mortality, individuals who died of undetermined cause were conservatively considered as not experiencing CV mortality and were censored at their respective date of death. For the examination of baseline smoking status and mortality, never smokers and former smokers were grouped together as nonsmokers and were compared against current smokers. Only patients who survived to discharge were included in the analyses. Multivariable Cox proportional hazards models incorporated adjustment for all baseline covariates that had a statistically significant (2-sided *P* ≤ .05) univariate association with the outcome in question.

To investigate the consequences of cessation, patients in the persistent smoking group were compared against those in the cessation group. Individuals were excluded from the analysis if death occurred within 1 year after the date of the index MI so as to prevent immortal time bias from the extended exposure period.^44^ Survival free from the outcomes of interest was then examined by means of a 1-year landmark analysis. For each outcome, multivariable Cox proportional hazards models adjusted for the propensity score on a continuous basis, in addition to variables not included in the propensity score but that had statistically significant univariate association (2-sided *P* ≤ .05) with the outcome in question. Details of the sensitivity analyses are available in eAppendix 4 in the Supplement.

## Results

### Baseline Characteristics

The cohort consisted of 2072 individuals (median age, 45 years [interquartile range, 42-48 years]; 1669 [80.6%] male) with baseline smoking status available who experienced an MI. Among them, 703 (33.9%) were never smokers, 281 (13.6%) were former smokers, and 1088 (52.5%) were current smokers at the time of their index hospitalization. When examining the baseline characteristics of the cohort, nonsmokers and smokers had a similar demographic makeup (Table 2); however, smokers had a higher NSS7 and were less likely to have private insurance. Smokers were also more likely to have presented with an ST-elevation MI (STEMI), to be diagnosed as having depression or psychotic disorder, to be nonobese, to have either alcohol use or illicit substance use, to have lower high-density lipoprotein cholesterol levels, and to undergo coronary revascularization. Other baseline characteristics are listed in Table 2.

**Table 2.** Baseline Characteristics of the Study Population Stratified by Smoking Status at the Index Myocardial Infarction (MI)

Variable	No./total No. (%)		*P* value
	Nonsmokers (n = 984)	Smokers (n = 1088)	
**Demographic characteristics**			
Age at the time of MI, median (IQR), y	46 (41 to 48)	45 (42 to 48)	.34
Male sex	808 (82.1)	861 (79.1)	.09
White race	707 (71.8)	809 (74.4)	.20
Neighborhood stress score, median (10%, 25%, 75%, 90%)	−0.82 (−1.29, −1.12, −0.03, 0.31)	−0.52 (−1.17, −0.94, 0.00, 0.83)	<.001
Insurance category^a^			
None	73/921 (7.9)	99/1026 (9.6)	.06
Public	260/921 (28.2)	325/1026 (31.7)	
Private	588/921 (63.8)	602/1026 (58.7)	
History of premature CAD in a first-degree relative	263 (26.7)	317 (29.1)	.22
ST-elevation MI	458 (46.5)	651 (59.8)	<.001
Segment Involvement Score, median (IQR)	2 (1 to 4)	3 (1 to 4)	.23
Index LVEF, median (IQR), %	56 (48 to 62)	55 (45 to 61)	.16
Depression	105 (10.7)	154 (14.2)	.02
Anxiety	125 (12.7)	153 (14.1)	.36
Psychotic disorder	17 (1.7)	44 (4.0)	.002
Length of index hospital stay, median (IQR), d	3 (2 to 6)	3 (2 to 5)	.15
**Risk factors**			
Diabetes	206 (20.9)	207 (19.0)	.28
Hypertension	475 (48.3)	495 (45.5)	.21
Hyperlipidemia	892 (90.7)	998 (91.7)	.39
Obesity	397/950 (41.8)	377/1055 (35.7)	.005
Alcohol use	62/967 (6.4)	207/1077 (19.2)	<.001
Illicit substance use	66 (6.7)	166 (15.3)	<.001
Angina	862/965 (89.3)	952/1063 (89.6)	.87
Peripheral vascular disease	15 (1.5)	26 (2.4)	.16
ASCVD score, median (10%, 25%, 75%, 90%)	3.4 (0.9, 1.9, 5.3, 9.0)	7.1 (3.1, 4.7, 11.1, 17.6)	<.001
Charlson Comorbidity Index score, mean (SD)	1.6 (1.1)	1.5 (1.0)	.32
**Laboratory values, median (10%, 25%, 75%, 90%)**			
Total cholesterol, mg/dL	187 (137, 159, 218, 259)	186 (132, 157, 220, 247)	.18
HDL cholesterol, mg/dL	37 (27, 31, 43, 52)	35 (25, 29, 41, 48)	<.001
LDL cholesterol, mg/dL	116 (69, 89, 144, 175)	116 (68, 91, 141, 168)	.67
Triglycerides, mg/dL	143 (70, 98, 214, 348)	153 (82, 107, 226, 347)	.02
Creatinine, mg/dL	1.0 (0.8, 0.9, 1.2, 1.4)	1.0 (0.7, 0.9, 1.1, 1.3)	<.001
eGFR, mL/min/1.73 m^2^	87.4 (60.2, 72.1, 101.3, 108.4)	90.3 (64.1, 77.0, 103.9, 109.4)	<.001
**Patient management**			
Cardiac catheterization	919 (93.4)	1029 (94.6)	.26
Coronary revascularization	783 (79.6)	929 (85.4)	<.001
Coronary artery bypass grafting	111 (11.3)	65 (6.0)	<.001
**Medical therapy at discharge**^**b**^			
Statin	836/963 (86.8)	974/1069 (91.1)	.002
Statin intensity			
None	127/963 (13.2)	95/1069 (8.9)	.003
Low/unknown dose	31/963 (3.2)	38/1069 (3.6)	
Moderate	321/963 (33.3)	421/1069 (39.4)	
High	484/963 (50.3)	515/1069 (48.2)	
Ezetimibe	14/963 (1.5)	12/1069 (1.1)	.51
Aspirin	895/963 (92.9)	1025/1069 (95.9)	.004
P2Y12 inhibitor	745/963 (77.4)	914/1069 (85.5)	<.001
β-Blocker	865/963 (89.8)	990/1069 (92.6)	.03
ACE inhibitor/ARB	577/963 (59.9)	674/1069 (63.0)	.15
Diuretic	121/963 (12.6)	99/1069 (9.3)	.02

Abbreviations: ACE, angiotensin-converting enzyme; ARB, angiotensin II receptor blocker; ASCVD, atherosclerotic cardiovascular disease; CAD, coronary artery disease; eGFR, estimated glomerular filtration rate; HDL, high-density lipoprotein; IQR, interquartile range; LDL, low-density lipoprotein; LVEF, left ventricular ejection fraction.

SI conversion factors: To convert cholesterol level to millimoles per liter, multiply by 0.0259; creatinine level to micromoles per liter, multiply by 88.4; and triglycerides level to millimoles per liter, multiply by 0.0113.

^a^ For the 1947 patients (94.0%) with available insurance data.

^b^ For 2032 patients (963 nonsmokers and 1069 smokers) who survived to discharge.

### Outcomes Stratified by Smoking at the Index Admission

#### All-Cause Mortality

Over a median follow-up of 11.2 years (interquartile range, 7.3-14.2 years), 142 of 1069 smokers (13.3%) in the cohort died compared with 87 of the 963 nonsmokers (9.0%) (*P* = .08). The mortality rate among patients identified as smokers was 1.10 deaths per 100 person-years, whereas the mortality rate among patients identified as never smokers or former smokers was 0.91 deaths per 100 person-years (*P* = .19). Smoking at the time of the index MI had an unadjusted HR of 1.21 (95% CI, 0.91-1.59; *P* = .19) (eFigure 1A in the Supplement). After adjusting for available covariates, the HR remained non–statistically significant (HR, 1.27; 95% CI, 0.90-1.79; *P* = .17) (eFigure 1A in the Supplement).

#### CV Mortality

Over the same median follow-up period, smokers had a similar prevalence of CV mortality compared with individuals who were nonsmokers at the time of their index hospitalization (50 of 1069 [4.7%] vs 36 of 963 [3.7%]; *P* = .32). The CV mortality rate among current smokers was 0.45 deaths per 100 person-years, whereas the CV mortality rate for never smokers and former smokers was 0.38 deaths per 100 person-years (*P* = .50). The unadjusted HR for CV mortality was 1.16 (95% CI, 0.75-1.79; *P* = .50) (eFigure 1B in the Supplement). After adjusting for available covariates, the HR remained non–statistically significant (HR, 1.10; 95% CI, 0.68-1.79; *P* = .69) (eFigure 1B in the Supplement).

### Smoking Cessation

Among the 1053 patients who were smokers at the index admission and survived longer than 365 days, complete data on smoking status were available for 910 (86.4%), as shown in eFigure 3 in the Supplement. Of them, 343 (37.7%) had quit smoking (the cessation group), whereas the remaining 567 (62.3%) continued smoking tobacco after their index admission (the persistent smoking group). There was no change in the rate of smoking cessation over the study period (eFigure 2 in the Supplement). Individuals who continued to smoke were similar to those who stopped smoking in terms of both age at the time of MI and race. Also, there was no difference in low-density lipoprotein cholesterol level reduction or low-density lipoprotein cholesterol level achieved at 1 year after MI between individuals who quit vs those who continued to smoke (eTable 1 in the Supplement). In contrast, individuals who continued to smoke after MI were more likely to have a higher NSS7, be diagnosed as having depression or psychotic disorder, have illicit substance use, and smoke more cigarettes per day, but they were less likely to have extensive CAD. Notably, those who stopped smoking were more likely to have private insurance than those who continued to smoke after their MI. The full set of patient characteristics is listed in Table 3.

**Table 3.** Baseline Characteristics, Patient Management, and Medical Therapy of Individuals Who Were Smokers at the Time of Their Myocardial Infarction (MI) Stratified by Whether They Quit or Continued to Smoke at 1-Year Follow-up

Variable	No./total No. (%)		*P* value
	Cessation group (n = 343)	Persistent smoking group (n = 567)	
**Demographic characteristics**			
Age at the time of MI, median (IQR), y	45 (42 to 48)	45 (41 to 48)	.87
Male sex	280 (81.6)	436 (76.9)	.09
White race	253 (73.8)	428 (75.5)	.56
Neighborhood stress score, median (10%, 25%, 75%, 90%)	−0.66 (−1.18, −0.99, 0.00, 0.70)	−0.44 (−1.16, −0.92, 0.06, 0.92)	.002
Insurance category^a^			
None	33/327 (10.1)	52/535 (9.7)	.02
Public	85/327 (26.0)	187/535 (35.0)	
Private	209/327 (63.9)	296/535 (55.3)	
History of premature CAD in a first-degree relative	102 (29.7)	180 (31.7)	.53
ST-elevation MI	192 (56.0)	351 (61.9)	.08
Segment Involvement Score, median (IQR)	3 (2 to 4)	2 (1 to 4)	.009
Index LVEF, median (IQR), %	56 (48 to 62)	55 (46 to 62)	.25
Depression	39 (11.4)	103 (18.2)	.006
Anxiety	47 (13.7)	93 (16.4)	.27
Psychotic disorder	6 (1.7)	28 (4.9)	.01
Length of index hospital stay, median (IQR), d	3 (2 to 5)	3 (2 to 5)	.22
Packs per day			
Median (IQR)	1.0 (0.8 to 1.5)	1.0 (1.0 to 1.5)	.04
Mean (SD)	1.1 (0.7)	1.2 (0.7)	.10
**Risk factors**			
Diabetes	64 (18.7)	111 (19.6)	.73
Hypertension	150 (43.7)	261 (46.0)	.50
Hyperlipidemia	320 (93.3)	519 (91.5)	.34
Obesity	152 (44.3)	184 (32.5)	<.001
Alcohol use	56 (16.3)	123 (21.7)	.05
Illicit substance use	39 (11.4)	99 (17.5)	.01
Angina	301 (87.8)	508 (89.6)	.17
Peripheral vascular disease	11 (3.2)	10 (1.8)	.16
ASCVD score, median (10%, 25%, 75%, 90%)	7.2 (3.2, 4.8, 10.1, 18.2)	7.3 (3.0, 4.6, 11.9, 17.6)	.86
Charlson Comorbidity Index score, mean (SD)	1.4 (0.7)	1.6 (1.2)	.04
**Laboratory values, median (10%, 25%, 75%, 90%)**			
Total cholesterol, mg/dL	187 (137, 161, 224, 247)	187 (130, 157, 220, 251)	.56
HDL cholesterol, mg/dL	35 (26, 30, 40, 48)	35 (25, 29, 41, 48)	.92
LDL cholesterol, mg/dL	118 (68, 91, 143, 166)	117 (68, 93, 140, 171)	>.99
Triglycerides, mg/dL	158 (85, 108, 248, 378)	154 (82, 108, 222, 355)	.47
Creatinine, mg/dL	1.0 (0.8, 0.9, 1.1, 1.3)	0.9 (0.7, 0.8, 1.1, 1.2)	.09
eGFR, mL/min/1.73 m^2^	90.5 (64.5, 78.2, 103.5, 108.5)	91.1 (66.5, 77.6, 104.9, 11.7)	.48
**Patient management**			
Cardiac catheterization	333 (97.1)	533 (94.0)	.04
Coronary revascularization	309 (90.1)	477 (84.1)	.01
Coronary artery bypass grafting	31 (9.0)	34 (6.0)	.08
**Medical therapy at discharge**			
Statin	321 (93.6)	513 (90.5)	.10
Statin intensity			
None	22 (6.4)	54 (9.5)	.23
Low/unknown dose	10 (2.9)	20 (3.5)	
Moderate	122 (35.6)	213 (37.6)	
High	189 (55.1)	280 (49.4)	
Ezetimibe	3 (0.9)	8 (1.4)	.47
Aspirin	333 (97.1)	544 (95.9)	.37
P2Y12 inhibitor	300 (87.5)	484 (85.4)	.37
β-Blocker	326 (95.0)	518 (91.4)	.04
ACE inhibitor/ARB	221 (64.4)	349 (61.6)	.38
Diuretic	34 (9.9)	53 (9.3)	.78
Reduction in LDL cholesterol achieved at 1 y after MI, median (10%, 25%, 75%, 90%), mg/dL	40 (−21, 7, 73, 95)	36 (−29, 2, 69, 115)	.53
% Change in LDL cholesterol achieved, mean (SD)	27.5 (34.5)	19.9 (52.6)	.17

Abbreviations: ACE, angiotensin-converting enzyme; ARB, angiotensin II receptor blocker; ASCVD, atherosclerotic cardiovascular disease; CAD, coronary artery disease; eGFR, estimated glomerular filtration rate; HDL, high-density lipoprotein; IQR, interquartile range; LDL, low-density lipoprotein; LVEF, left ventricular ejection fraction.

SI conversion factors: To convert cholesterol level to millimoles per liter, multiply by 0.0259; creatinine level to micromoles per liter, multiply by 88.4; and triglycerides level to millimoles per liter, multiply by 0.0113.

^a^ For the 862 patients (94.7%) with available insurance data.

A comparison of the baseline characteristics between current smokers for whom follow-up data on smoking status at 1 year after MI were available (n = 910) and those without any follow-up data (n = 143) is summarized in eTable 2 in the Supplement. There were no statistically significant differences in the incidence of either all-cause mortality (HR, 1.02; 95% CI, 0.60-1.74; *P* = .95) or CV mortality (HR, 0.82; 95% CI, 0.38-1.78; *P* = .62) between individuals for whom follow-up data were present vs individuals for whom follow-up data were absent.

### Outcomes Stratified by Smoking Cessation After MI

#### All-Cause Mortality

**Figure 1.**
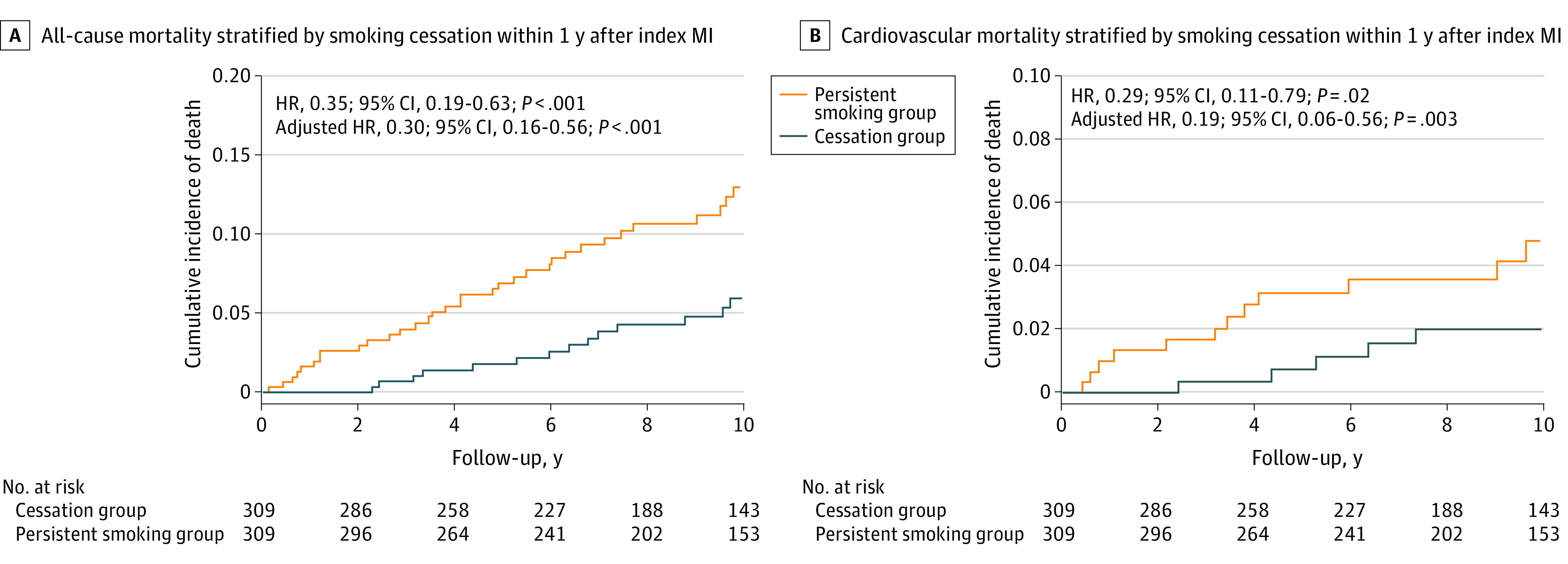
All-Cause and Cardiovascular Mortality by Smoking Cessation After Myocardial Infarction (MI) Shown are Kaplan-Meier failure curves of all-cause and cardiovascular mortality stratified by smoking cessation after MI. HR indicates hazard ratio.

Over a median follow-up of 10.2 years (interquartile range, 7.0-13.1 years), 75 of 567 individuals (13.2%) in the persistent smoking group had died compared with 14 of 343 individuals (4.1%) in the cessation group (*P* < .001). Within the matched sample, the unadjusted HR of smoking cessation was 0.35 (95% CI, 0.19-0.63; *P* < .001) (Figure 1A). After adjustment using the propensity score, as well as additional adjustment for diabetes, hypertension, peripheral vascular disease, CCI score, estimated glomerular filtration rate, and P2Y12 inhibitor use at discharge, the HR of all-cause mortality was 0.30 (95% CI, 0.16-0.56; *P* < .001). These results remained similarly robust after incorporating pack-years into the propensity score model (eAppendix 3 in the Supplement).

#### CV Mortality

Compared with individuals who quit smoking, those who smoked tobacco after their MI had higher CV mortality (30 of 567 [5.3%] vs 6 of 343 [1.7%]; *P* = .008). In the matched sample, the unadjusted HR for smoking cessation was 0.29 (95% CI, 0.11-0.79; *P* = .02) (Figure 1B). After adjustment using the propensity score, as well as additional adjustment for history of premature CAD in a first-degree relative, length of index hospital stay, diabetes, hypertension, peripheral vascular disease, estimated glomerular filtration rate, and P2Y12 inhibitor and β-blocker use at discharge, the HR of CV mortality was 0.19 (95% CI, 0.06-0.56; *P* = .003). As with all-cause mortality, these results remained similarly robust after including pack-years in the propensity score model (eAppendix 3 in the Supplement).

### Combined Analysis

**Figure 2.**
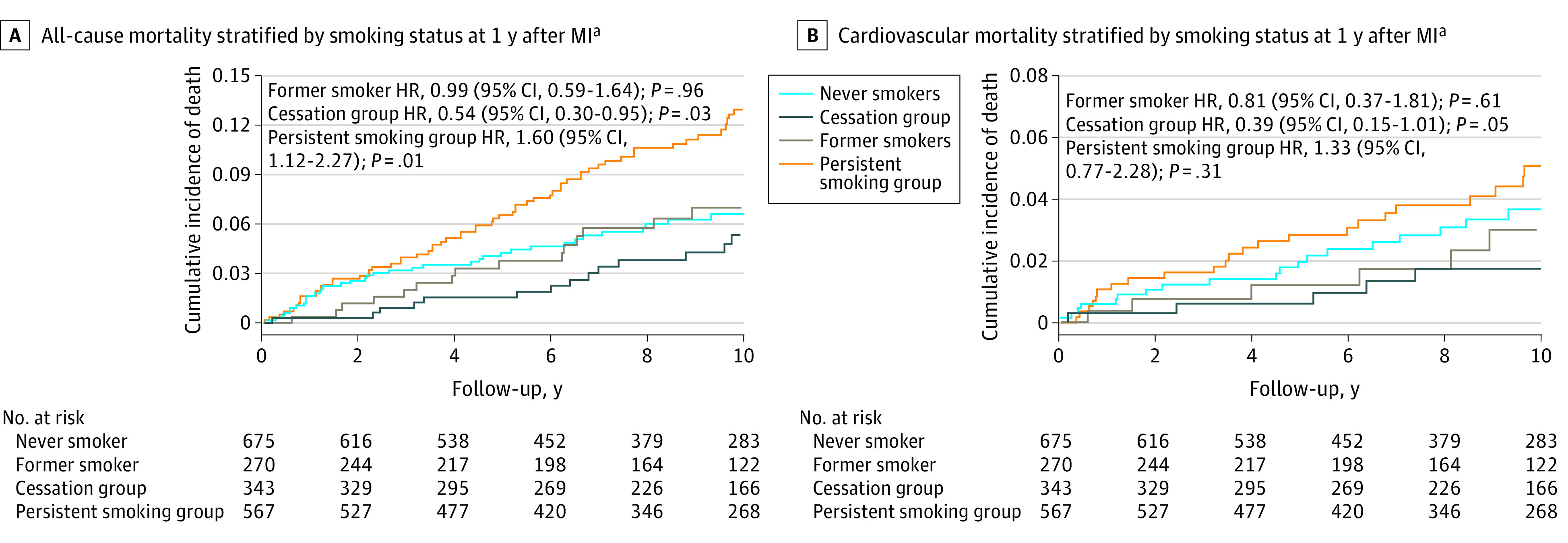
All-Cause and Cardiovascular Mortality by Smoking Status After Myocardial Infarction (MI) Shown are Kaplan-Meier failure curves of all-cause and cardiovascular mortality stratified by smoking status at 1 year after MI. ^a^Unadjusted HRs reported.

Using a 1-year landmark analysis, this study compared the relative risk of all-cause and CV mortality across all 4 subgroups in the sample population (never smokers, former smokers, cessation group, and persistent smoking group). Using never smokers as the referent group, the unadjusted HRs for all-cause mortality were 0.99 (95% CI, 0.59-1.64; *P* = .96) for former smokers, 0.54 (95% CI, 0.30-0.95; *P* = .03) for the cessation group, and 1.60 (95% CI, 1.12-2.27; *P* = .01) for the persistent smoking group (Figure 2A). Similarly, for CV mortality, the HRs were 0.81 (95% CI, 0.37-1.81; *P* = .61) for former smokers, 0.39 (95% CI, 0.15-1.01; *P* = .05) for the cessation group, and 1.33 (95% CI, 0.77-2.28; *P* = .31) for the persistent smoking group (Figure 2B). After adjusting for available covariates, there were no statistically significant changes in the ranking of the relative risk or the associated level of statistical significance for either outcome (Figure 2A and B).

### Sensitivity Analysis

Two separate analyses were performed to assess the sensitivity of the findings relative to potential missing confounders. The results remained unchanged (eAppendix 3 in the Supplement).

## Discussion

Among 2072 patients who experienced an MI at a young age, 52.5% were current smokers at the time of their index hospitalization. Approximately 62% of those who were smokers at the time of their MI continued to smoke. Although patients who quit smoking were similar to those who continued to smoke with respect to their baseline characteristics, smoking cessation was associated with an approximate 70% to 80% reduction in all-cause and CV mortality. These findings suggest similar or even greater association of decreased mortality with smoking cessation in young individuals compared with prior research^12,24,45,46,47^ and highlight the critical importance of smoking cessation, especially in the post-MI setting. The similar risk burden between patients who quit smoking and those who continued to smoke suggests that smoking cessation likely has substantial consequences on reducing the risk of mortality after MI.

To our knowledge, this study is the first to exclusively examine the association of smoking and smoking cessation with the long-term outcomes of young individuals who have experienced an MI. The mean age of both our full cohort and the subset of patients classified as smokers at their MI is at least 5 years younger^25,45,47,48^ and more than 10 years younger^13,15,18,20,21,23,24,26,27,28^ than the cohorts of previous studies. Therefore, our findings not only substantiate previous research on the benefits of smoking cessation in general, but also support them in individuals who experience an MI at a young age.

Focusing our analysis on a younger population presented certain advantages as well. First, we were able to avoid the smoker’s paradox because both the nonsmokers and current smokers were approximately the same age and had similar baseline risk profiles. In addition, given the early age of presentation, the possible benefits of smoking cessation in follow-up may be even more substantial.

When comparing the mortality outcomes for all 4 subgroups of patients in our cohort, there were 2 noteworthy findings. First, the long-term mortality rates of never smokers and those individuals who had quit smoking before their incident MI (ie, former smokers) were almost identical. This finding is in line with prior work demonstrating that the CV risk attributable to smoking attenuates substantially after sustained cessation.^18,49^ Accordingly, our work supports this literature by confirming that prolonged smoking cessation reduces the risk from prior smoking to near baseline levels in a population of patients who experienced an MI at a young age.

A second finding is that patients who quit smoking after their index MI had the lowest long-term mortality rates, with HRs approximately 40% to 60% lower than the referent never smoker group. This fact was at least partly responsible for the lack of observed statistically significant differences when examining the association of baseline smoking with long-term prognosis. For those individuals who were smokers at the time of their MI, smoking may have been the primary risk factor that predisposed them to the event. Prior research from large epidemiologic studies^50,51,52,53^ has found that current smoking increases the risk of incident MI between 3-fold and 6-fold. In this young population of patients who likely had a limited chronological exposure to other conventional risk factors (eg, hypertension, hyperlipidemia, etc) because of their young age, current tobacco use may have been the primary instigator of their MI. Once this key risk factor was withdrawn through smoking cessation, these individuals had a statistically significantly lower risk burden. In contrast, those individuals who were never smokers or former smokers likely had an unmeasured predisposition to CV disease—genetic or otherwise—that is not modifiable. This hypothesis would explain our finding that individuals who quit smoking after their MI had lower long-term mortality compared with the referent never smoker group and warrants further investigation.

Finally, smokers were more likely to undergo coronary revascularization than never smokers and former smokers and to be treated with statins and antiplatelet agents (Table 2), which is possibly associated with the fact that they could have had more severe disease, as is also suggested by their higher prevalence of STEMI. However, the benefit of these therapies could have been attenuated by other factors that adversely altered their prognosis, such as higher NSS7s and higher illicit substance use.

### Clinical Implications

Although the importance of smoking cessation counseling is recognized by multiple guidelines and public health measures, more than half of the cohort were smokers at the time of their MI, with roughly 60% of those continuing to smoke after their MI.^54,55^ The proportion of those who continued to smoke after the initial MI is surprisingly high considering that individuals are generally more likely to quit smoking after a substantial medical event.^56,57,58^ The observed rate of persistent smokers herein is also high compared with that observed in prior studies.^11,18,20,21^ Although our cohort’s share of smokers at the time of the index admission was not substantially different from past studies^12,15,20,25,27,28^ focused on older populations, the proportion of individuals who continued to smoke after their MI is, with the exception of a few cases,^24,46,59^ at least 5% higher^9,13,15,22,45,48^ and sometimes more than 10% higher^10,11,12,14,16,17,18,20,21,47^ than that observed in prior research. Therefore, not only is smoking prevalent among individuals who experience an MI, but also younger patients may be less likely to quit than their older counterparts.

The results of this study demonstrate the potential magnitude of the health benefits that may be achieved over an intermediate period through smoking cessation. Furthermore, because the findings are limited to mortality and thus do not include other nonfatal events, such as cerebrovascular accidents, MIs, repeat hospitalizations, revascularizations, or new cancer diagnoses, the overall benefits of cessation are likely much higher.

### Limitations

This study has some limitations. Given our study’s retrospective design, the independent association of smoking cessation with mortality in young patients with MI may be overestimated because of unmeasured confounding factors, such as healthy lifestyle and protective behavioral factors. However, offsetting this limitation was our ability to examine more than 2000 individuals who experienced an initial MI at a young age, which would have been challenging to accomplish given the overall low prevalence of such events on a population level.

An additional limitation was our time-invariant definition of smoking cessation. By limiting cessation to 1 year after MI, we do not account for either those individuals who may have ceased smoking at a later point after their MI or the possibility that individuals who stopped smoking may have relapsed at a later date. However, because each of these scenarios would, in effect, bias our results toward the null, limiting cessation to within 1 year of the index MI results in a conservative measure of the association of smoking cessation and long-term mortality.

Our study population was also limited to those individuals who had an MI. As such, it was not possible to examine the effect size of smoking on the incidence of MI among younger individuals. That limitation notwithstanding, the data in this study indicate that smoking cessation is associated with statistically significantly improved long-term mortality in this patient population.

Given the retrospective nature of this study, we relied on self-reported smoking cessation, as documented in the electronic medical records. Although an objective measure of smoking cessation, such as urinary cotinine testing, would have been ideal, our rate of continued smoking is similar—if not higher—than that reported in other studies.^11,18,20,21,24^ This study obtained vital status data from state and national death repositories. Although these databases are largely complete, it is possible that an individual’s death may have occurred but was not reported.

A final limitation of our study was the inability to obtain follow-up data regarding smoking status at 1 year for all patients who were identified as tobacco users during their index MI. However, when we conducted a sensitivity analysis comparing patients for whom data were available with those patients for whom either 1-year smoking status or propensity score data were missing, there were no statistically significant differences in the rates of either all-cause or CV mortality (eAppendix 5 in the Supplement). These results suggest that excluding these patients from the analysis is unlikely to have biased our results regarding the benefits of smoking cessation.

## Conclusions

Almost fifty-three percent of patients who experienced an initial MI at 50 years or younger in this retrospective study cohort were smokers. Smoking cessation after the incident MI was associated with a 70% decreased risk of mortality compared with those who continued smoking at 1 year after their MI. These findings reinforce the critical importance of smoking cessation, especially among those who experience an MI at a young age.

## Notes

Supplement. **eAppendix 1.** Definition of Risk Factors **eAppendix 2.** Description of Propensity Score **eAppendix 3.** Propensity Score Model With Pack-Years **eAppendix 4.** Sensitivity Analysis **eAppendix 5.** Comparison of Patients Based on Availability of Tobacco Cessation Data **eTable 1.** Change in LDL and LDL Achieved Stratified by Smoking Cessation at 1 Year After MI **eTable 2.** Comparison of Baseline Characteristics for Patients With and Without 1-Year Smoking Status **eFigure 1.** Kaplan-Meier Failure Curves of All-Cause Mortality and Cardiovascular Mortality Stratified by Smoking Status at Index Myocardial Infarction **eFigure 2.** Trend in Smoking Cessation **eFigure 3.** Flow Chart of Patients **eReferences.**
