# Supplementary material for: Association of Smoking Cessation and Survival Among Young Adults With Myocardial Infarction in the Partners YOUNG-MI Registry
Source: JAMA Netw Open. 2020 Jul 8;3(7):e209649. doi: 10.1001/jamanetworkopen.2020.9649 (PMC7344383; doi:10.1001/jamanetworkopen.2020.9649)
Supplement: Supplement. — eAppendix 1. Definition of Risk Factors eAppendix 2. Description of Propensity Score eAppendix 3. Propensity Score Model With Pack-Years eAppendix 4. Sensitivity Analysis eAppendix 5. Comparison of Patients Based on Availability of Tobacco Cessation Data eTable 1. Change in LDL and LDL Achieved Stratified by Smoking Cessation at 1 Year After MI eTable 2. Comparison of Baseline Characteristics for Patients With and Without 1-Year Smoking Status eFigure 1. Kaplan-Meier Failure Curves of All-Cause Mortality and Cardiovascular Mortality Stratified by Smoking Status at Index Myocardial Infarction eFigure 2. Trend in Smoking Cessation eFigure 3. Flow Chart of Patients eReferences. [file jamanetwopen-3-e209649-s001.pdf]

Supplementary Online Content

Biery DW, Berman AN, Singh A, et al. Association of smoking cessation and survival among young adults with myocardial infarction in the Partners YOUNG-MI registry. *JAMA Netw Open*. 2020;3(7):e209649. doi:10.1001/jamanetworkopen.2020.9649

eAppendix 1. Definition of Risk Factors

eAppendix 2. Description of Propensity Score

eAppendix 3. Propensity Score Model With Pack-Years

eAppendix 4. Sensitivity Analysis

eAppendix 5. Comparison of Patients Based on Availability of Tobacco Cessation Data

eTable 1. Change in LDL and LDL Achieved Stratified by Smoking Cessation at 1 Year After MI

eTable 2. Comparison of Baseline Characteristics for Patients With and Without 1-Year Smoking Status

eFigure 1. Kaplan-Meier Failure Curves of All-Cause Mortality and Cardiovascular Mortality Stratified by Smoking Status at Index Myocardial Infarction

eFigure 2. Trend in Smoking Cessation

eFigure 3. Flow Chart of Patients

eReferences.

This supplementary material has been provided by the authors to give readers additional information about their work.

© 2020 Biery DW. *JAMA Network Open*.

eAppendix 1. Definition of Risk Factors

Diabetes was defined as having a fasting plasma glucose >126 mg/dL, a hemoglobin A1c ≥6.5%, or a documented diagnosis and/or treatment for diabetes. Hypertension was defined as a documented diagnosis and/or treatment of hypertension. Dyslipidemia was defined as having a documented diagnosis and/or treatment of dyslipidemia. Obesity was defined as having a body mass index (BMI) ≥30 kg/m^2 or a documented diagnosis of obesity. Family history of premature CAD was defined as a fatal MI, a non-fatal MI, or a coronary revascularization occurring before 55 years of age in the case of first-degree male family members and before 65 years of age for first-degree female family members. Alcohol use was defined as having a positive toxicology report for alcohol during the index hospitalization or a documented diagnosis and/or treatment of alcohol use. Illicit substance use was defined as having a positive toxicology report during the index hospitalization for one or more of marijuana, cocaine, heroin, or methamphetamines or a documented diagnosis and/or treatment for use of these substances. Depression, anxiety, and psychotic disorders were defined as having a documented diagnosis and/or treatment for these conditions. In addition, we also calculated the atherosclerotic cardiovascular disease (ASCVD) risk score for each individual whenever possible.[1]

To adjust for comorbidities, we calculated the Charlson Comorbidity Index, a method of predicting the risk of mortality based on co-morbidities of patients, for each patient as determined by International Classification of Diseases, 9th Revision (ICD-9) diagnosis and billing codes associated with the index hospitalization.[2] For patients within the sample who were not coded for MI, we added an additional ICD-9 code of “410” (acute MI) prior to calculating the CCI. Additionally, because lower socioeconomic

© 2020 Biery DW. *JAMA Network Open*.

status is known to be associated with all-cause death, household income for each patient was estimated based on household zip codes in conjunction with the 2015 inflation adjusted median household income data provided by the US Census Bureau.[3]

Invasive angiographic findings were ascertained through a review of all available cardiac catheterization images by a single interventional cardiologist blinded to all patient outcomes and risk factors. A segment involvement score (SIS) was employed as a measure of the extent of plaque and was calculated as the total number of coronary artery segments exhibiting plaque, irrespective of the degree of luminal stenosis within each segment. For each patient, the SIS ranged from a minimum of 0 to a maximum of 16.

© 2020 Biery DW. *JAMA Network Open*.

eAppendix 2. Description of Propensity Score

The independent variables included in the score were age, sex, neighborhood stress score (NSS7) type of insurance, segment involvement score, presence of an ST-elevation MI (STEMI), depression, psychotic disorders, obesity, alcohol use, cocaine use, cardiac catheterization, coronary revascularization, as well as statins, and beta-blockers upon discharge.

Of the 910 patients with a smoking status at one-year post-MI, we were able to calculate a propensity score for 876 (96%). The loss of 34 observations was due solely to the absence angiographic data on those individuals. Due to the relatively small number of missing observations, they were handled by means of simple deletion. Among these, 336 (38%) had ceased smoking tobacco, while the remaining 540 (62%) continued to smoke. We matched 309 (92%) of those individuals who quit smoking post-MI to an individual who continued to smoke. There was significant overlap in the propensity scores between patients who quit smoking and those who did not. Patients who ceased tobacco smoking post-MI had a mean propensity score of 0.43 (95% CI 0.41 to 0.44), while those who continued to smoke had a mean propensity score of 0.35 (95% CI 0.34 to 0.36). Balance in the propensity score was assessed by means of the stratification method by means of the PSCORE command.[4] Specifically, the full range of the propensity score was first split into 5 equally spaced intervals. Within each interval, the average propensity score of the treated (i.e. those who quit smoking) was compared to the average propensity score of the control (i.e. those who continued to smoke) to ensure that there was no difference.

© 2020 Biery DW. *JAMA Network Open*.

eAppendix 3. Propensity Score with Pack-Years

Data on pack-years was available for 718 (66%) of patients identified as current smokers at the time of their index admission and 632 (69%) of smokers with an available smoking status at one-year post-MI. Of these, we were able to calculate a propensity score for 607 (96%), with 238 (39%) having ceased tobacco smoking and the remaining 369 (61%) having continued to smoke. We matched 203 (85%) of those individuals who quit smoking post-MI to an individual who continued to smoke. Within the matched sample, the unadjusted hazard ratio of smoking cessation on all-cause mortality was 0.33 (CI 0.15 to 0.73, p=0.006). After adjusting for the propensity score on a continuous basis, the adjusted hazard ratio was 0.19 (CI 0.044 to 0.87, p=0.033). Within the same matched sample, the unadjusted hazard ratio of smoking cessation on cardiovascular mortality was 0.27 (CI 0.11 to 0.66, p=0.004). After adjusting for the propensity score on a continuous basis, the adjusted hazard ratio was 0.10 (CI 0.02 to 0.73, p=0.023).

© 2020 Biery DW. *JAMA Network Open*.

eAppendix 4. Sensitivity Analysis

We used two methods to assess the validity of our findings. First, we determined how sensitive the results of the model were to the individual removal of each of the included covariates. The goal of doing so would be to mimic the effect of an influential yet unmeasured confounder on the stability of our model. The relative hazard of all-cause death changed by an absolute maximum of 0.12 when any single variable was removed but continued to be significant in each scenario. The relative hazard of CV death changed by an absolute maximum of 0.14 when any single variable was removed but, as with all-cause mortality, remained significant. Second, we repeated the analysis of smoking cessation after weighting by the inverse probability of treatment (IPTW) to account for any possible bias in self-selection for smoking cessation and, hence, a discrepancy between the average treatment effect on the treated (ATT) and the average treatment effect (ATE) of smoking cessation.[5,6] Stabilized weights[6] were used to reduce the influence of outliers on estimates of the effect of smoking cessation on the outcome in question.[6,7]After weighting, the adjusted hazard ratios of smoking cessation on all-cause death and CV death were 0.26 (CI 0.12 to 0.54, $p<0.001$) and 0.19 (CI 0.05 to 0.69, $p=0.01$), respectively. Error in the estimation of the weights was assumed to be negligible. Moreover, the robust sandwich-type variance estimator was used as it would provide the most conservative measure of significance.[8]

© 2020 Biery DW. *JAMA Network Open*.

eAppendix 5. Comparison of Patients Based on Availability of Tobacco Cessation Data

Of the 1053 individuals determined to be current smokers at the time of their MI and who survived for more than one year following their index infarction, smoking status at one-year post-MI was unavailable for 143. Differences in the baseline characteristics between the those for which data was present (n=910) and those for which it was absent (n=143) are presented in Supplemental Table 2. Those patients for which no follow-up data were available at one-year post-MI were less likely to be diagnosed with psychotic disorders or depression, less likely to be obese, less likely to use alcohol. Despite these differences, there was no significant difference in all-cause mortality, with hazard ratio for no follow-up of 1.02 (CI 0.60 to 1.74), p=0.95. Similarly, for cardiovascular mortality the hazard ratio for no follow-up was 0.82 (CI 0.38 to 1.78), p=0.62. Similar results were found after including those patients for which we were unable to calculate a propensity score.

© 2020 Biery DW. *JAMA Network Open*.

eTable 1. Change in LDL and LDL Achieved stratified by Smoking Cessation at One Year Post-MI.

**Factor**	**Quit Smoking (n=343, 38%)**	**Cont. Smoking (n=567, 62%)**	**p-value**
Absolute Change in LDL-Cholesterol, median (IQR)	-40 (-7, -73)	-36 (-2, -69)	0.53
Percentage Change in LDL-Cholesterol, median (IQR)	-35 (-7, -52)	-27 (-4, -49)	0.21
LDL Achieved at One-Year Post-MI, median (IQR)	82 (64, 103)	88 (71, 111)	0.058

© 2020 Biery DW. *JAMA Network Open*.

eTable 2. Comparison of baseline characteristics for current smokers with and without one-year smoking status

Baseline Variable	Smoking Status Post-MI Available (n=910)	Smoking Status Post-MI Unavailable (n=143)	p-value
**Demographics**			
Age, median (IQR)	45 (41, 48)	45 (42, 48)	0.68
Female Sex	194 (21.3%)	27 (18.9%)	0.51
Caucasian	681 (74.8%)	105 (73.4%)	0.72
Income, median (IQR)	64.2 (50.9, 82.0)	66.5 (52.1, 84.5)	0.35
Insurance Category			
None	85 (9.9%)	10 (7.5%)	0.50
Public	272 (31.6%)	39 (29.1%)	
Private	505 (58.6%)	85 (63.4%)	
Premature CAD in 1st Degree Relative	282 (31.0%)	30 (21.0%)	0.015
ST-Elevation Myocardial Infarction	543 (59.7%)	84 (58.7%)	0.83
Depression	142 (15.6%)	8 (5.6%)	0.001
Anxiety	140 (15.4%)	8 (5.6%)	0.002
Psychotic Disorders	34 (3.7%)	5 (3.5%)	0.89
Length of Stay, median (IQR)	3.0 (2.0, 5.0)	3.0 (3.0, 5.0)	0.19
Packs Per Day, median (IQR)	1.0 (1.0, 1.5)	1.0 (1.0, 1.5)	0.15
**Risk Factors**			
Hypertension	411 (45.2%)	64 (44.8%)	0.93
Dyslipidemia	839 (92.2%)	130 (90.9%)	0.60
Diabetes Mellitus	175 (19.2%)	22 (15.4%)	0.27
Obesity	336 (38.0%)	30 (21.9%)	<0.001
Alcohol Use	179 (19.8%)	17 (12.2%)	0.033
Illicit Substance Use	138 (15.2%)	17 (11.9%)	0.30
Angina	809 (91.0%)	117 (90.0%)	0.67
Peripheral Vascular Disease (PVD)	21 (2.3%)	2 (1.5%)	0.55
ASCVD Score, median (IQR)	7.2 (4.6, 11.2)	6.7 (4.8, 10.5)	0.97
Charlson Comorbidity Index, median (IQR)	1.0 (1.0, 2.0)	1.0 (1.0, 2.0)	0.11
**Laboratory Values**			
Total Triglycerides mg/dL, median (IQR)	155 (108, 228)	141 (107, 206)	0.20
Total Cholesterol mg/dL, mean (SD)	192.1 (58.0)	182.5 (42.3)	0.076
HDL Cholesterol mg/dL, mean (SD)	35.7 (9.1)	35.4 (11.3)	0.72
LDL Cholesterol mg/dL, mean (SD)	119.8 (51.1)	113.2 (33.9)	0.17
Estimated Glomerular Filtration Rate (eGFR) mL/min/1.73 m^2, mean (SD)	90.3 (18.9)	89.5 (16.7)	0.63
Creatinine mg/dL, median (IQR)	1.0 (0.8, 1.1)	1.0 (0.9, 1.1)	0.52
**Patient Management**			
Cardiac Catheterization	866 (95.2%)	135 (94.4%)	0.70
Coronary Revascularization	786 (86.4%)	119 (83.20%)	0.31
**Medical Therapy**			
Aspirin at Discharge	877 (96.4%)	132 (92.3%)	0.024
Statin at Discharge	834 (91.6%)	128 (89.5%)	0.40
P2Y12 Inhibitor at Discharge	784 (86.2%)	115 (80.4%)	0.071
Beta Blocker at Discharge	844 (92.7%)	131 (91.6%)	0.63
ACE Inhibitor / ARBs at Discharge	570 (62.6%)	94 (65.7%)	0.48
Diuretic at Discharge	87 (9.6%)	9 (6.3%)	0.21
**Outcomes**			
All-Cause Mortality	90 (9.9%)	16 (11.2%)	0.65
Cardiovascular Death	34 (3.7%)	8 (5.6%)	0.35

© 2020 Biery DW. *JAMA Network Open*.

eFigure 1. Kaplan Meier Failure Curves of All-Cause Mortality and Cardiovascular Mortality Stratified by Smoking Status at Index Myocardial Infarction

A. Kaplan Meier Failure Curves of All-Cause Mortality Stratified by Smoking Status at Index Myocardial Infarction

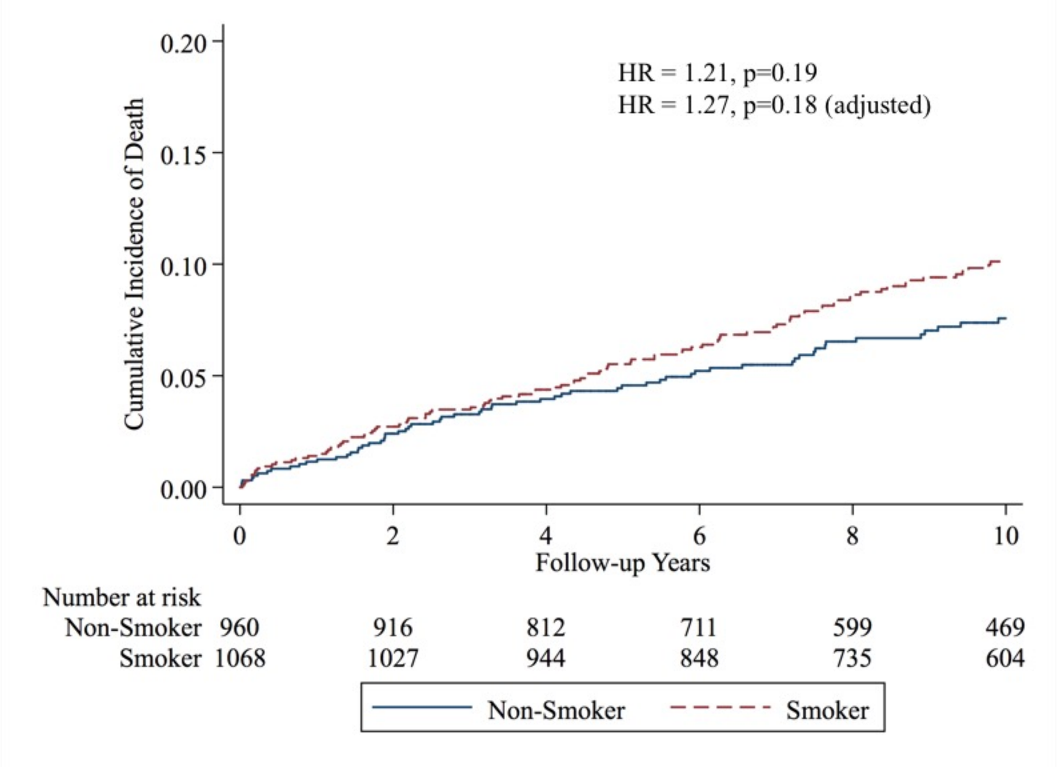

© 2020 Biery DW. *JAMA Network Open*.

B. Kaplan Meier Failure Curves of Cardiovascular Mortality Stratified by Smoking Status at Index Myocardial Infarction

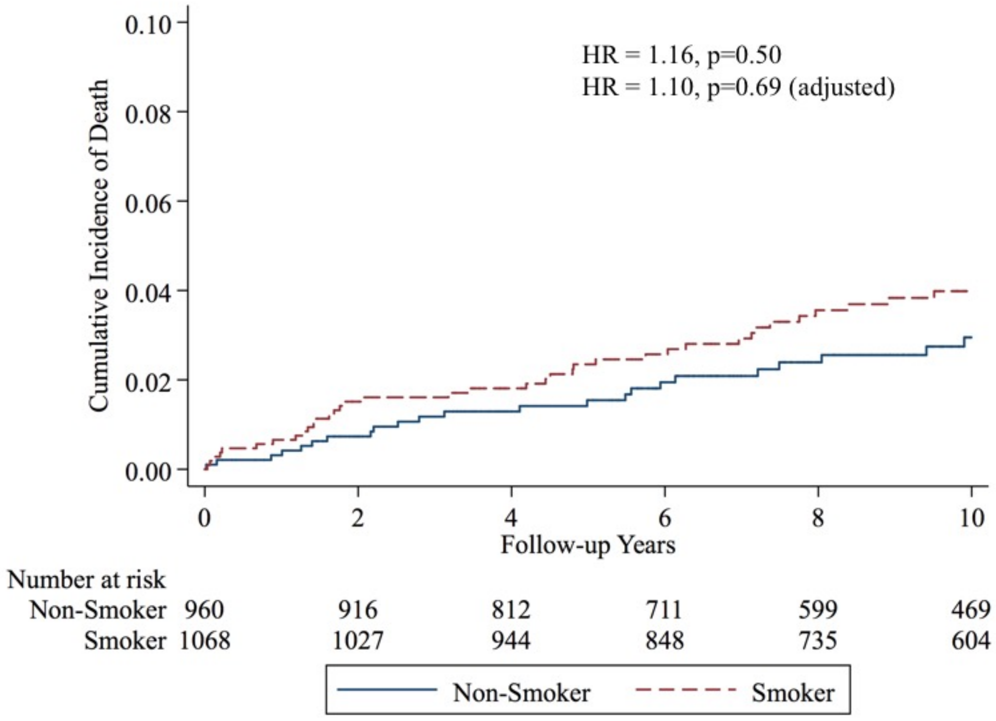

© 2020 Biery DW. *JAMA Network Open*.

eFigure 2. Trend in Smoking Cessation

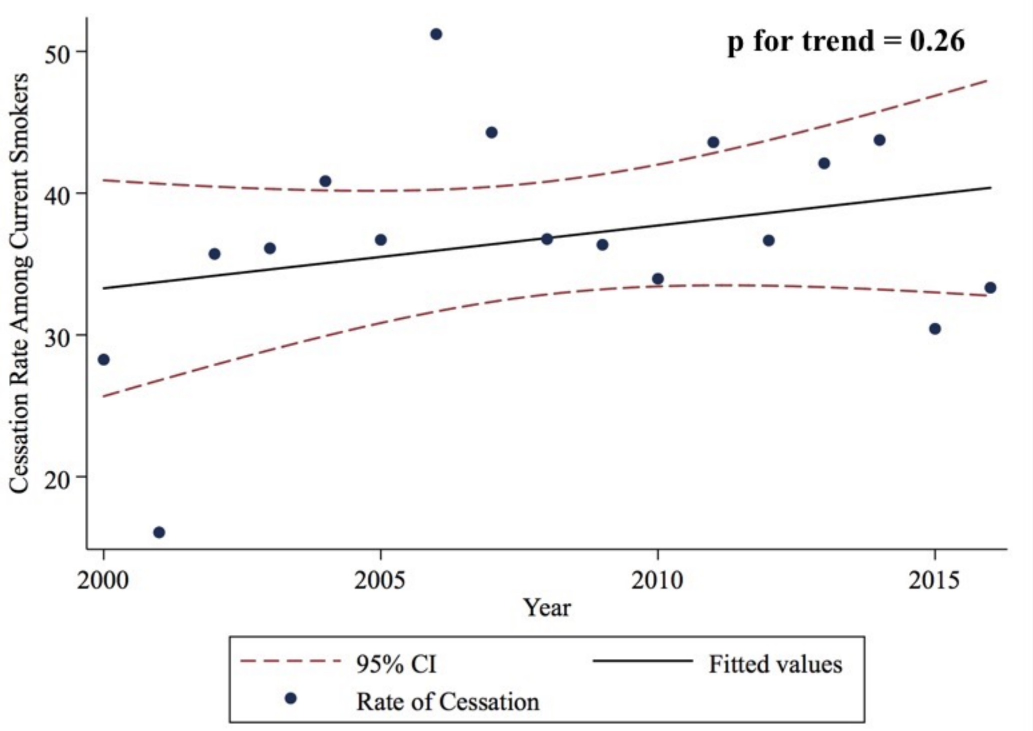

© 2020 Biery DW. *JAMA Network Open*.

eFigure 3. Flow Chart of Patients

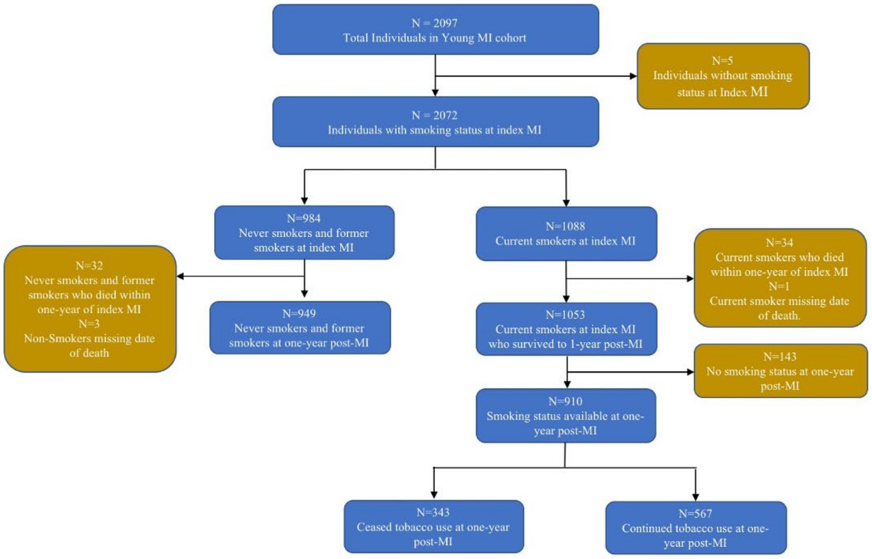

© 2020 Biery DW. *JAMA Network Open*.

eReferences

1. Singh A, Collins BL, Gupta A, et al. Cardiovascular Risk and Statin Eligibility of Young Adults After an MI: Partners YOUNG-MI Registry. *Journal of the American College of Cardiology.* 2018;71(3):292-302.
2. *CHARLSON: Stata module to calculate Charlson index of comorbidity* [computer program]. 2017.
3. U.S. Census Bureau. Median Household Income in the Past 12 Months (in 2015 inflation-adjusted dollars). In: U.S. Census Bureau; 2015.
4. Becker SO, Ichino A. Estimation of Average Treatment Effects Based on Propensity Scores. *The Stata Journal.* 2002;2(4):358-377.
5. Austin PC. The relative ability of different propensity score methods to balance measured covariates between treated and untreated subjects in observational studies. *Medical decision making : an international journal of the Society for Medical Decision Making.* 2009;29(6):661-677.
6. Hernan MA, Brumback B, Robins JM. Marginal structural models to estimate the causal effect of zidovudine on the survival of HIV-positive men. *Epidemiology (Cambridge, Mass).* 2000;11(5):561-570.
7. Schafer JL, Kang J. Average causal effects from nonrandomized studies: a practical guide and simulated example. *Psychological methods.* 2008;13(4):279-313.
8. Austin PC. Variance estimation when using inverse probability of treatment weighting (IPTW) with survival analysis. *Stat Med.* 2016;35(30):5642-5655.

© 2020 Biery DW. *JAMA Network Open*.
